# Adrenal cavernous hemangioma: a case report

**DOI:** 10.1186/s12893-018-0429-9

**Published:** 2018-11-20

**Authors:** Carlo V. Feo, Alessandro De Troia, Massimo Pedriali, Simone Sala, Maria Chiara Zatelli, Paolo Carcoforo, Claudio F. Feo

**Affiliations:** 1 Department of Surgery, Unit of General Surgery, Azienda USL di Ferrara, and University of Ferrara, Ferrara, Italy; 2 Department of Surgery, Unit of Surgery 2, S. Anna University Hospital of Ferrara, and University of Ferrara, Ferrara, Italy; 3 Department of Diagnostic Imaging and Laboratory Medicine, Unit of Anatomic Pathology, S. Anna University Hospital of Ferrara, and University of Ferrara, Ferrara, Italy; 4 Department of Diagnostic Imaging and Laboratory Medicine, Unit of Radiology, S. Anna University Hospital of Ferrara, Ferrara, Italy; 5 Department of Medical Sciences, Section of Endocrinology and Internal Medicine, S. Anna University Hospital of Ferrara, and University of Ferrara, Ferrara, Italy; 6 Department of Clinical and Experimental Medicine, Unit of General Surgery 2, University of Sassari, Sassari, Italy; 7 UO di Chirurgia Generale Provinciale, Azienda USL di Ferrara, Via Valle Oppio, 2, Room 1.210 44023 Lagosanto, Ferrara, Italy

**Keywords:** Cavernous hemangioma, Incidentaloma, Adrenal gland, Adrenal tumor, Adrenalectomy

## Abstract

**Background:**

Adrenal cavernous hemangiomas are very rare benign tumors that usually present as incidental findings on abdominal imaging. Preoperative differential diagnosis from other benign or malignant adrenal neoplasms may be challenging.

**Case presentation:**

A 70-year old man was referred for an 8-cm abdominal mass incidentally discovered on a contrast-enhanced computed tomography (CT) performed to investigate a pulmonary nodule. Biochemical tests ruled out any endocrine dysfunction and iodine 123 metaiodobenzylguanidine whole body scintiscan single-photon emission CT excluded a pheocromocitoma. Findings on magnetic resonance imaging were non-specific and the patient was elected for a left adrenalectomy. Histopathological diagnosis revealed a cavernous hemangioma. A portion of the resected tissue was tested for drug sensitivity to mitotane, doxorubicin, and sunitinib.

**Conclusions:**

Adrenal hemangioma is a rare disease but should be included in the differential diagnosis of adrenal tumors. The surgical resection is generally required to exclude malignant disease, resolve pressure-related symptoms, and prevent retroperitoneal hemorrhage. Although specific features in diagnostic imaging are often lacking, if the diagnosis is established preoperatively a laparoscopic adrenalectomy can be performed due to the benign nature of the lesion. Doxorubicin and sunitinib were both capable of reducing primary culture cell viability, this suggest that similar drugs may be useful in the medical treatment of adrenal hemangiomas.

## Background

Adrenal cavernous hemangioma is a rare entity first described in the mid-1950 [1]. Cavernous hemangioma most commonly affects the skin and liver, it is mainly discovered incidentally on radiographic imaging, and the definitive diagnosis is usually postoperative.

We report the case of a non-functioning adrenal cavernous hemangioma incidentally discovered on a contrast-enhanced computed tomography (CT) and discuss the diagnostic work-up, surgical treatment, and post-operative findings including drug sensitivity testing.

## Case presentation

A 70-year-old man was referred to the S. Anna University Hospital in Ferrara (Italy) for a left upper quadrant abdominal mass incidentally discovered on a contrast-enhanced CT of the chest performed to investigate a 15-mm right pulmonary nodule.

The patient was asymptomatic, his past medical history was positive for essential hypertension, and physical examination was unremarkable.

**Fig. 1:**
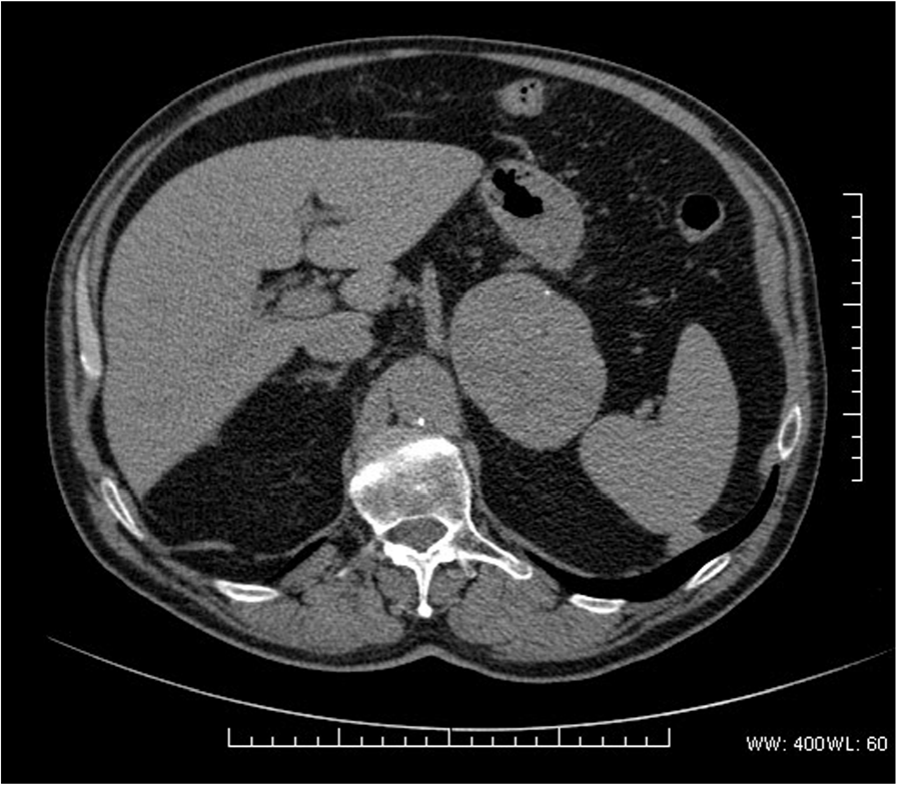
CT scan showing a large homogeneous lesion with rare peripheral calcifications

**Fig. 2:**
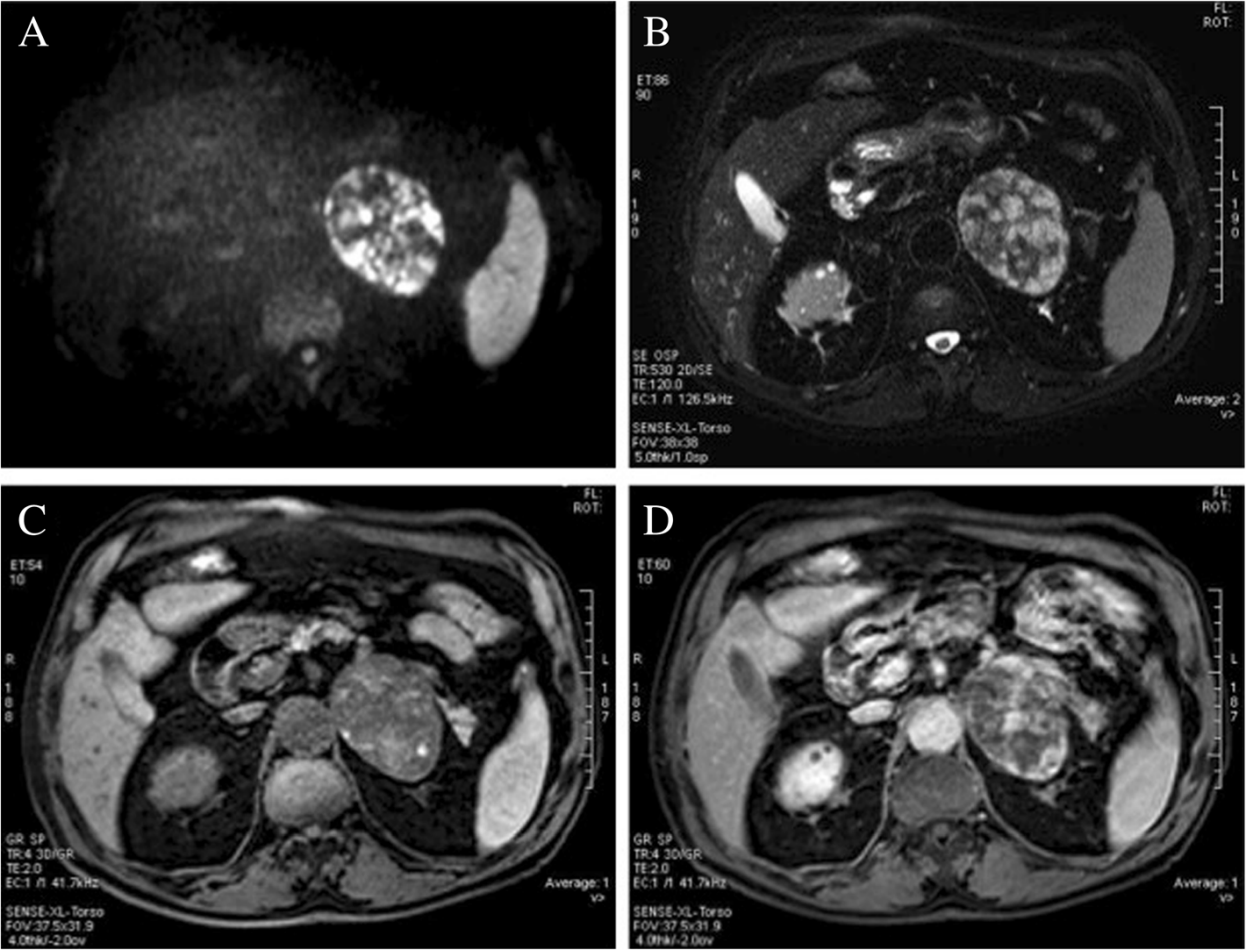
MRI shows restriction of intralesional molecular water diffusion in Diffusion Weighted Imaging (**a**), high signal intensity intralesional areas in T2 fat-saturated weighted image (**b**), high signal intensity intralesional areas in T1 fat-saturated weighted image (**c**) with inhomogeneous enhancement in contrast-enhanced T1 fat-saturated weighted image (**d**)

CT scan showed a homogeneous 83-mm left adrenal lesion with an average density of 45 HU; rare peripheral dot-like calcifications were also observed (Fig. 1). The right adrenal gland was normal. Due to high-density values of the left adrenal lesion excluding classic low-density adrenal adenoma, an abdominal magnetic resonance imaging (MRI) examination was subsequently performed. MRI with chemical shift imaging showed absence of signal intensity decrease in out-of-phase compared with in-phase images, restriction of intralesional molecular water diffusion in Diffusion Weighted Imaging (Fig. 2a) with high-intensity intralesional areas both in T1 and in T2 and T2 fat-saturated weighted images suggesting areas of intralesional subacute hemorrhage (Fig. 2b). After intravenous contrast medium administration of gadoteric acid (DOTAREM©, GUERBET S.p.A., Genova, Italy) at 0.1 mmol/kg, a thin capsular rim of early enhancement with slow heterogeneous centripetal enhancement was observed (Fig. 2c, d).

Biochemical tests ruled out any endocrine dysfunction (plasma renin 20,5 μU/ml, plasma aldosterone 7,6 ng/dl, urinary adrenaline 4.59 μg /24 h; urinary noradrenaline 43.35 pg/24 h, urinary metanephrine 120.75 μg/24 h, urine normetanephrine 250.25 μg/24 h). A subsequent iodine 123 metaiodobenzylguanidine whole body scintiscan single-photon emission computed tomography-CT (I123-MIBG-SPECT-CT) ruled out the presence of a pheocromocitoma.

Due to the non-specific radiological findings and the size of the lesion, a surgical resection was then elected to establish the final diagnosis. The patient underwent a left adrenalectomy trough a left subcostal incision. Intraoperatively, the mass appeared encapsulated and hypervascularised. No evidence of hepatic as well as other peritoneal lesions was present. The operation was straightforward and the postoperative course was uneventful, with the patient discharged home on postoperative day six.

**Fig. 3:**
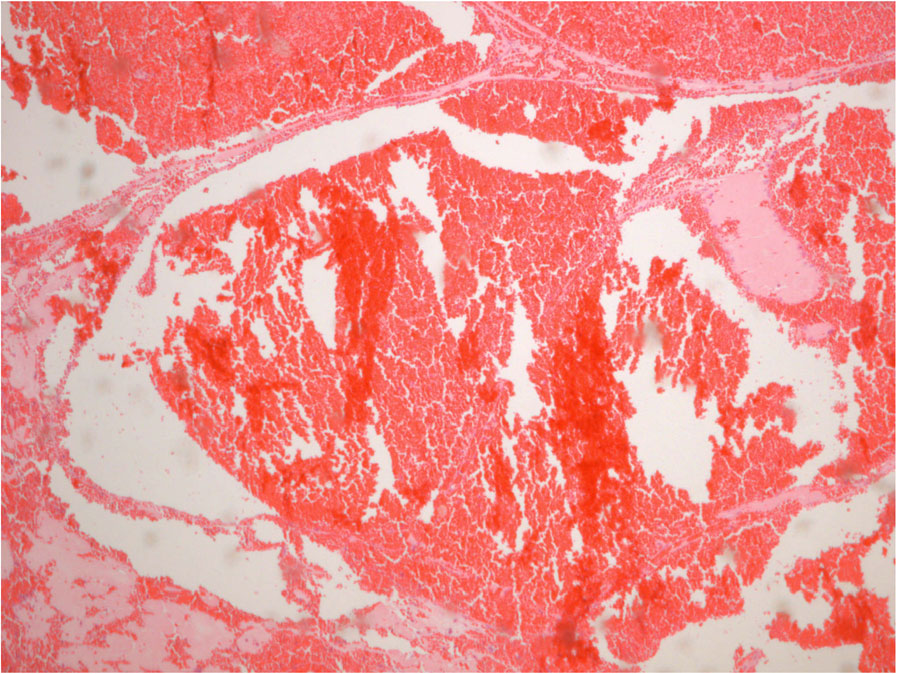
Hematoxilin and eosin stain (× 4) showing large vascular spaces lined by endothelial cells and separated by thick fibrous septa

The pathological examination revealed a large lesion of 90 mm × 65 mm × 70 mm with spongy appearance due to large vascular spaces. Histologically, the lesion showed a conglomerate of widely open vascular lumina lined by endothelial cells and separated by thick nearly acellular fibrous septa (Fig. 3). The final diagnosis of cavernous hemangioma was then made.

**Fig. 4:**
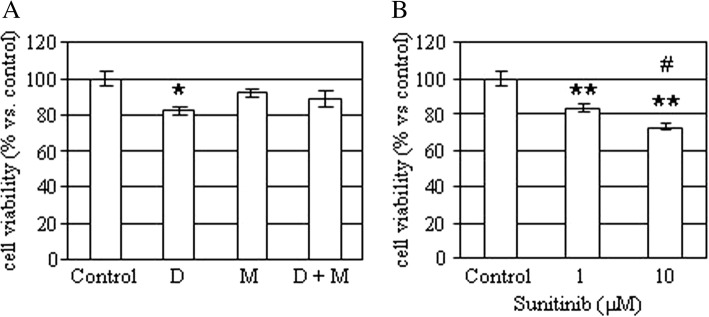
Effects of mitotane, doxorubicin and sunitinib on hemangioma primary culture. **a** Cells were incubated for 48 h with or without 50 nM doxorubicin (D), 5 μM mitotane (M), alone or in combination and then cell viability was measured. Data are expressed as the mean of six replicates ± SE. *P < 0.05 versus control untreated cells. **b** Cells were incubated for 48 h with or without 1 μM and 10 μM sunitinib and then cell viability was measured. Data are expressed as the mean of six replicates ± SE. **P < 0.01 versus control untreated cells. ^#^*P* < 0.5 versus doxorubicin-treated cells

A portion of the tissues was obtained at time of surgery and a primary culture was obtained, as described previously [2]. Cells were then incubated without or with 5 μM mitotane (an adrenolitic drug), 50 nM doxorubicin (a cytotoxic drug) or with 1–10 μM sunitinib (a VEGF inhibitor) and cell viability was assessed after 48 h, as previously described [3]. As shown in Figure 4, doxorubicin (− 18%; *p* < 0.05 vs. control), but not mitotane, was capable of reducing primary culture cell viability. Similarly, sunitinib significantly reduced cell viability both at 1 and at 10 μM (− 16% and − 27%, respectively; *p* < 0.01 vs. control).

At 53-month follow-up the patient is doing well and has no evidence of recurrence. However, he underwent a laparoscopic prosthetic repair of incisional, umbilical and left inguinal hernias 20 months following the adrenalectomy.

## Discussion and conclusions

Adrenal cavernous hemangiomas are rare benign tumors arising from endothelial cells lining the blood vessels that are usually discovered incidentally on abdominal imaging. They are unilateral in most cases, only one bilateral case has been reported in the literature [4], generally over 10 cm in diameter [5] which may reach up to 35 cm [6]. Adrenal hemangiomas generally present in the sixth to seventh decade of life and are more frequent in women with a male/female ratio of 2:1 [5]. In general they tend to be asymptomatic non-functioning tumors and only few secreting adrenal hemangiomas have been described in the literature [7]. Rarely, cavernous adrenal hemangiomas may become large [8] compressing surrounding structures and causing abdominal pain [6, 9] or may rupture leading to retroperitoneal hemorrhage [10–12].

Abdominal CT and MRI are valuable diagnostic tools for hemangiomas. On contrast-enhanced CT, the presence of both peripheral patchy and centripetal enhancements and highly dense peripheral rim are characteristic for adrenal hemangioma [13]. However, in the absence of centripetal enhancement the diagnosis is difficult as a thin-rim peripheral enhancement may be observed in other adrenal tumors. Although non-specific, MRI may show marked hyperintensity on T2-weighted images and focal hyperintensity on T1-weighted images as a consequence of bleeding and calcification that can guide to the correct diagnosis [13, 14]. Spotty calcifications throughout the tumor are probably due to phleboliths in dilated vascular spaces [11, 15] and are seen also in other adrenal tumors. Thus, the preoperative diagnosis of adrenal hemangioma remains very difficult [16] and the final diagnosis is largely established after surgical resection by the pathologist, as in this case. The in vitro data are in keeping with the vascular nature of the lesion. Indeed, mitotane, a well know adrenolytic drug, did not affect primary culture cell viability, which, on the contrary was reduced by a cytotoxic drug, such as doxorubicin. The evidence that sunitinib, a multitarget tyrosine kinase inhibitor, significantly reduced primary culture cell viability may suggest that this, or similar drugs, may be useful in the medical treatment of adrenal hemangiomas in patients who are not eligible for surgery and may take advantage of debulking medical therapy, although further studies are needed to support this hypothesis.

Surgical resection is indicated for enlarging adrenal tumors or suspected carcinoma, regardless of its endocrine activity [17]. Laparoscopic adrenalectomy is considered the standard of care for small to medium size benign adrenal lesions (i.e., less than 6–7 cm in diameter) [18]. Larger tumors, however, can be treated safely and effectively by a laparoscopic approach in experienced and specialized centers [19–22]. By contrast, an open adrenalectomy is indicated in case of suspected malignancy, although a laparoscopic approach may be attempted for 4 to 6-cm non-functioning tumors and conversion established in case of local invasion detected intraoperatively [17]. In the presented case, an open adrenalectomy was preferred due to the suspect of malignancy (hypervascularisation and calcifications on MRI) and the 9-cm size of the lesion.

In conclusion, the case of an asymptomatic 8-cm cavernous hemangioma incidentally discovered and successfully treated by open adrenalectomy is presented. Adrenal hemangioma is a rare disease but should be included in the differential diagnosis of adrenal tumors. The surgical resection is generally required to exclude malignant disease, resolve pressure-related symptoms, and prevent retroperitoneal hemorrhage. Although specific features in diagnostic imaging are often lacking, if the diagnosis is established preoperatively a laparoscopic adrenalectomy may be planned due to the benign nature of such a lesion.

## Abbreviations

CT: Computed tomography
MRI: Magnetic Resonance Imaging
